# Bcl-3 is a novel biomarker of renal fibrosis in chronic kidney disease

**DOI:** 10.18632/oncotarget.21692

**Published:** 2017-10-09

**Authors:** Ran Chen, Lunshan Wang, Sanhong Liu, Xi Chen, Yiming Hu, Hanshao Liu, Haohao Zhang, Yuhang Jiang, Qi Wang, Deji Ye, Lingling Li, Dandan Liu, Xiaorong Pan, Lixin Wei, Xuemei Li, Xiaoren Zhang

**Affiliations:** ^1^ Key Laboratory of Stem Cell Biology, Institute of Health Sciences, Shanghai Institutes for Biological Sciences, Chinese Academy of Sciences, Shanghai Jiao Tong University School of Medicine, Shanghai 200031, China; ^2^ Clinical Laboratory Department, The Chinese People’s Liberation Army 105th Hospital, Hefei 230001, China; ^3^ Shanghai Institute for Advanced Immunochemical Studies, ShanghaiTech University, Shanghai 201210, China; ^4^ Tumor Immunology and Gene Therapy Center, Eastern Hepatobiliary Surgery Hospital, The Second Military Medical University, Shanghai 200433, China; ^5^ Department of Nephrology, Peking Union Medical College Hospital, Chinese Academy of Medical Sciences, Peking Union Medical College, Beijing 100723, China

**Keywords:** Bcl-3, renal fibrosis, chronic kidney disease, unilateral ureteral obstruction, biomarker

## Abstract

Progressive renal fibrosis in chronic kidney disease (CKD) greatly contributes to end-stage renal failure and is associated with high mortality. The identification of renal fibrosis biomarkers for the diagnosis and the monitoring of disease progression in CKD is urgently needed. Whole-transcriptomic analysis of renal tissues in a unilateral ureteral obstruction (UUO) mouse model revealed that the mRNA level of Bcl-3, an atypical member of the IκB family, was induced 6.3-fold 2 days after UUO. Compared with renal tissues in sham-operated mice, increases in Bcl-3 mRNA and protein in the renal tissues in the UUO model were accompanied with increases in other markers of renal fibrosis, including human epididymis protein 4 (HE4), a recently identified biomarker of renal fibrosis. Immunohistochemical analysis revealed that both Bcl-3 and HE4 were located in the plasma of renal tubule cells. Serum protein levels of Bcl-3 and HE4 rose with the development of renal fibrosis in UUO mouse model. We found that the serum protein levels of both HE4 and Bcl-3 were elevated in CKD patients compared with healthy controls. Moreover, a significant positive correlation between Bcl-3 and HE4 (r = 0.939, p < 0.0001) was observed in CKD patients. These data suggest that Bcl-3 can serve as a novel valuable biomarker of renal fibrosis in CKD.

## INTRODUCTION

Chronic kidney disease (CKD) is characterized by the progressive deterioration of kidney function. The number of patients with CKD worldwide will double from 2010 to 2030 [1, 2]. In China, more than one million people die as a result of CKD and its complications annually. Renal fibrosis is a progressive process causing CKD, and this condition largely contributes to end-stage renal failure.

Renal fibrosis is a self-perpetuating process during which the deposition of type I collagen and other extracellular matrix proteins progressively replace the normal renal tissues, resulting in the structural and functional changes to the kidneys. Renal fibrosis is the consequence of deterioration caused by the recruitment of activated fibroblasts and the inflammatory responses that primarily result from acute and chronic renal injury. The TGFβ/Smad signaling pathway plays a critical role in the process of renal fibrosis [3, 4]. TGFβ promotes the epithelial-to-mesenchymal transition (EMT) of tubular epithelial cells (TECs) and induces various types of cells (such as mesangial cells, interstitial fibroblasts, and tubular epithelial cells) to differentiate into matrix-producing fibrogenic cells [5-7].

Currently, renal biopsy is still the established standard assessment to clarify the degree of kidney damage and renal fibrosis. Because of its invasive nature and associated risks, including hemorrhage, pain, and even death in a few patients, renal biopsy cannot be conducted serially [8]. Serum diagnostic biomarkers of renal fibrosis are still lacking. Human epididymis protein 4 (HE4), encoded by *Wfdc2*, is a putative pan-serine protease inhibitor. HE4 is elevated in the serum of CKD patients and is correlated with decreased kidney function and an advanced stage of renal fibrosis, suggesting that HE4 might be a valuable clinical biomarker of renal fibrosis in CKD [9, 10]. However, HE4 is increased in the sera of patients with cancers, such as ovarian cancers. Therefore, novel biomarkers for the detection and the monitoring of the progression of renal fibrosis in CKD are needed.

B cell lymphoma 3 (Bcl-3), a member of the IκB family, regulates gene transcription by interacting with NFκB1/p50 or NFκB2/p52 homodimer as a co-factor in the nucleus [11-14]. Bcl-3 is located in the cytoplasma and regulates ERK, AKT signaling [15, 16]. We recently reported that Bcl-3 regulates the TGFβ/Smad signaling pathway by directly binding to Smad3 and protecting the Smad3 protein from the ubiquitination and degradation. These data suggest that Bcl-3 might be involved in the renal fibrosis by regulating TGFβ signaling.

Here, we showed that the Bcl-3 mRNA and protein levels in the renal tissues of a unilateral ureteral obstruction (UUO) mouse model were significantly higher than those in sham-operation mice. The Bcl-3 protein level in the sera was increased in the CKD patients compared with that in the healthy controls and was significantly correlated with HE4 level in CKD patients. These data reveal that Bcl-3 could be a novel biomarker for the detection of renal fibrosis.

## RESULTS

### Differentially expressed genes of the NFκB signaling pathway in the UUO model were shown

**Figure 1:**
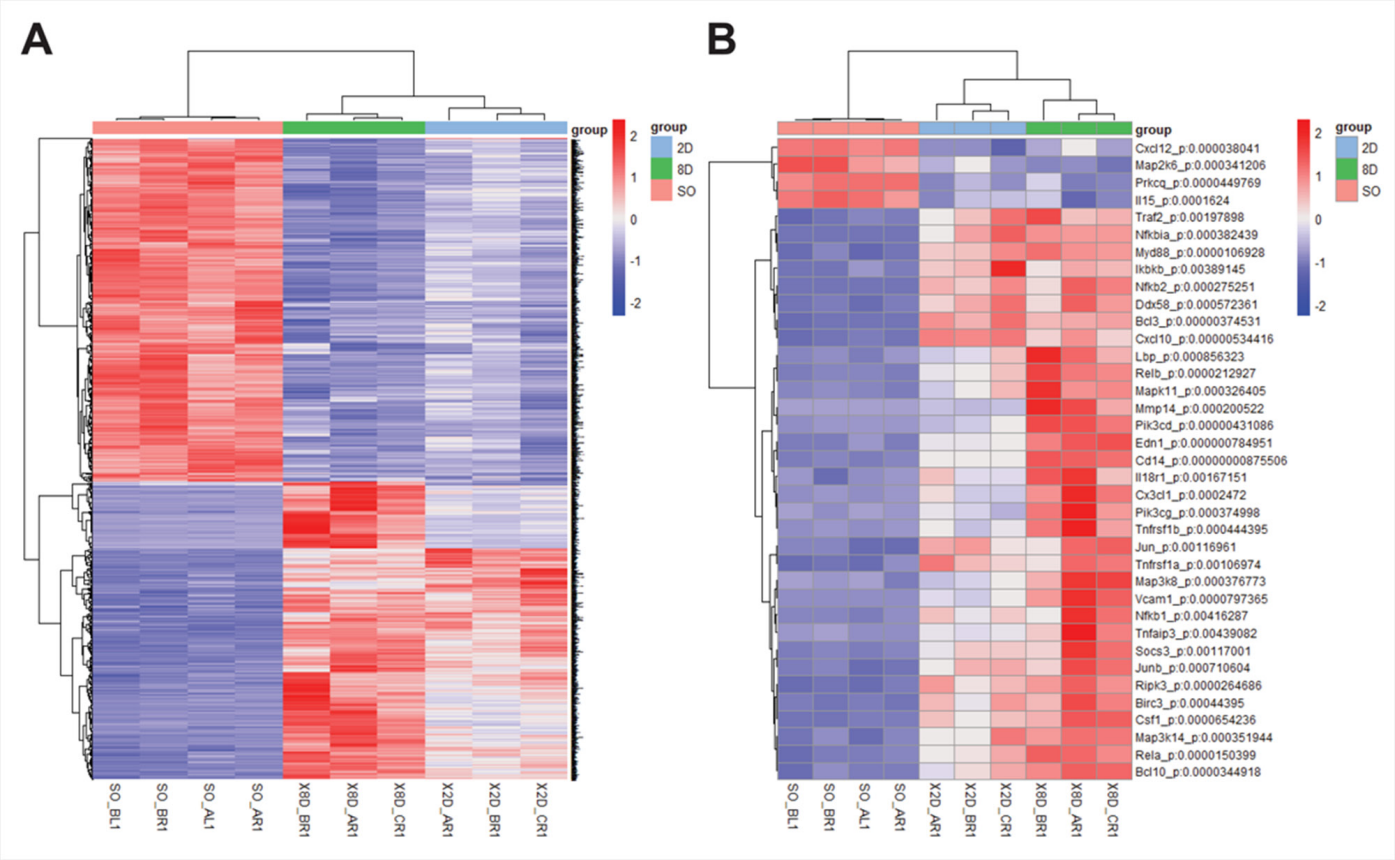
Differentially expressed genes of the NFκB signaling pathway in the UUO model were shown **(A)** Heatmap showing differentially expressed genes among sham-operated, 2-day ligated and 8-day ligated mice, expressed as the normalized and summed exonic read counts in the samples ( n=4 for sham-operated group, n=3 for 2-day ligated group and 8-day ligated group). **(B)** A total of 37 genes related to the NFκB pathway were detected in the enrichment analysis. The *P*-value was determined using two-way analysis of variance by Tukey’s multiple comparison test.

To find the biomarkers of renal fibrosis, we reanalyzed the existing data deposited in NCBI’s Gene Expression Omnibus, which were accessed through GEO Series accession number GSE79443 [17]. First, the differentially expressed genes were identified using statistical significance (p< 0.01), setting the cutoff values of up-regulation (>1.5-fold) and down-regulation (>1.5-fold) compared to the baseline. Using these parameters, 1736 genes were identified (Figure 1A). Given that both NFκB signaling and TGFβ signaling contribute to the process of renal fibrosis we focused on the NFκB signaling related genes [18]. Using DVAID enrichment analysis (https://david.ncifcrf.gov/), 37 genes related to the NFκB signaling were identified as differentially expressed (Figure 1B). We have previously shown that Bcl-3 regulates the TGFβ signaling and hypothesized that Bcl-3 might be involved in renal fibrosis. Expectedly, the expression of *Bcl-3* mRNA was significantly higher in the UUO model.

### The *Bcl-3* mRNA level was increased in the renal tissues at 15 days after UUO

**Figure 2:**
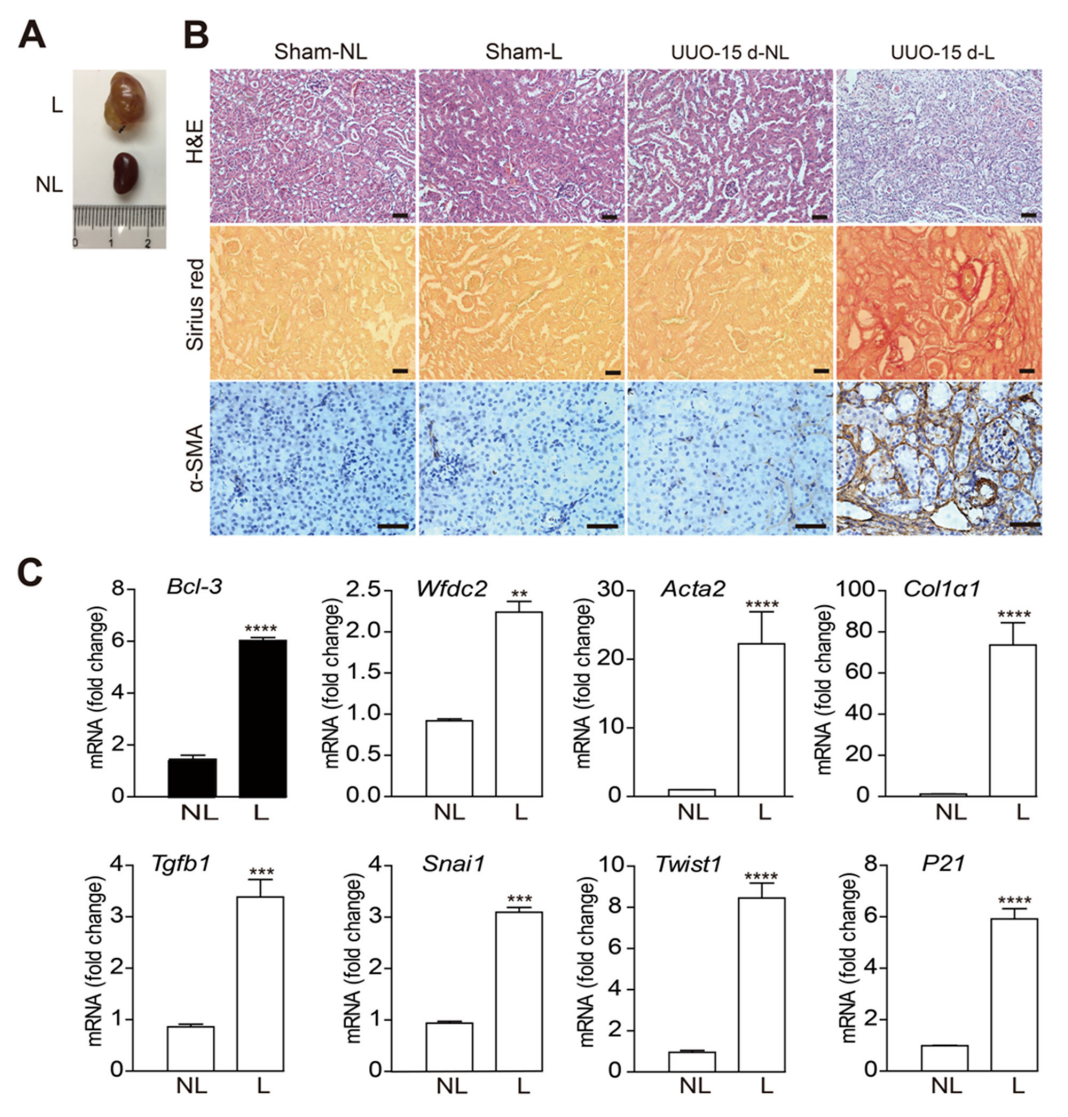
The *Bcl-3* mRNA level was increased in renal tissues 15 days after UUO **(A)** Unilateral ureteral obstruction was maintained for 15 days in WT C57bl/6 mice, and the mice were euthanized 15 days later. **(B)** Representative images of overall morphology using H&E, Sirius red staining and α-SMA immunohistochemistry. Scale bars, 50 μm. **(C)** *Bcl-3, Wfdc2, Snai1, Acta2, Col1α1, Tgfb1, P21* and *Twist1* mRNA levels were detected using RT-PCR. All data are expressed as the means ± s.e.m. ^*^P < 0.05, ^**^P < 0.01, ^***^P < 0.001 and ^****^P < 0.0001 were determined using Student’s t-test. A total of 3 individuals were included in each sham group, and 5 individuals were included in each UUO group.

To validate the expression of NFκB signaling-related genes, we conducted unilateral ureteral obstruction (UUO) in C57bl/6 wild-type (WT) mice, a standard model for progressive renal fibrosis [19, 20]. Ureteral ligation resulted the urine retention and kidney deterioration after 15 days (Figure 2A). The overall morphology of the kidneys was analyzed using in the H&E staining, collagen deposition was analyzed using the Sirius red staining, and α-smooth muscle actin expression was analyzed using immunohistochemistry. The results showed organic disorder and the accumulation of many necrotic-cell fragments, as previously described [21]. We also observed increased α-SMA+ cells and collagen deposition in obstructed kidneys at 15 days after UUO surgery (Figure 2B). These changes revealed that the UUO mouse model was successfully performed.

The mRNA levels of NFκB signaling-related genes and the genes encoding fibrotic markers [22] were detected using RT-PCR in obstructed (L) and in contralateral non-obstructed kidneys (NL) at 15 days after UUO or sham operation (Figure 2C). Similar to the mRNA levels of other fibrotic markers, *Bcl-3* mRNA levels were markedly up-regulated at 15 days after surgery (Figure 2C).

### The *Bcl-3* mRNA level persistently increased from 1 day after UUO

**Figure 3:**
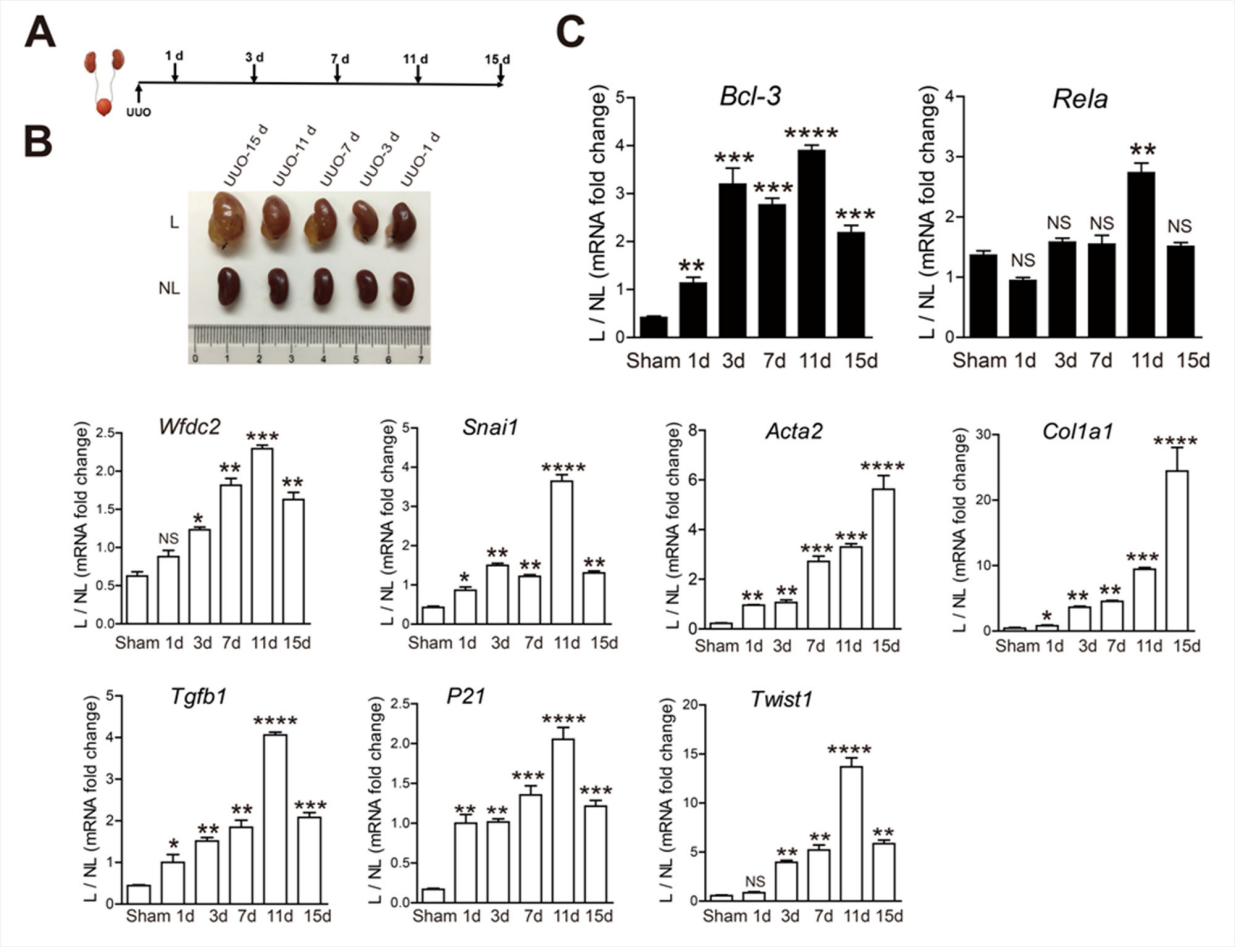
The *Bcl-3* mRNA level persistently increased from 1 day after UUO **(A)** Schematic of the experimental approach. UUO was conducted in WT C57bl/6 mice, which were sacrificed 1, 3, 7, 11 and 15 days after obstruction. **(B)** L and NL kidneys from UUO mice are shown at 1, 3, 7, 11 and 15 days after obstruction. **(C)** *Bcl-3*, *Rela*, *Wfdc2, Snai1*, *Acta2, Col1α1, Tgfb1, P21* and *Twist1* mRNA levels of kidneys from sham and UUO mice were measured at 5 different time points using RT-PCR. All data are expressed as the means ± s.e.m. ^*^P < 0.05, ^**^P < 0.01, ^***^P < 0.001 and ^****^P < 0.0001 were determined using Student’s t-test. A total of 3 individuals were included in each sham group, and 5 individuals were included in each UUO group.

To examine the dynamical expression of *Bcl-3, Rela* and the fibrotic marker genes, such as *Wfdc2, Snai1, Acta2, Col1α1, Tgfb1, P21* and *Twist1*, in UUO-induced fibrosis, UUO was conducted in mice for 1, 3, 7, 11 or 15 days (Figure 3A). These mice progressively developed different levels of CKD and renal fibrosis. Slight urine retention was detected in the kidneys at 1 day after UUO, and symptoms of renal fibrosis were barely observed (Figure 3B). The mRNA levels of many genes, including *Acta2, Col1α1, Tgfb1, P21* and *Bcl-3*, were increased after 1 day after UUO (Figure 3C). The expression of *Wfdc2* increased from 3 days after UUO (Figure 3C). Compared with controls, the *Bcl-3* mRNA levels showed a persistently significant increase from the beginning of UUO-induced renal fibrosis (Figure 3C). These data indicated that *Bcl-3* mRNA levels increased earlier than *Wfdc2*.

### The Bcl-3 protein level in fibrotic kidneys showed a significant increase from 3 days after UUO

**Figure 4:**
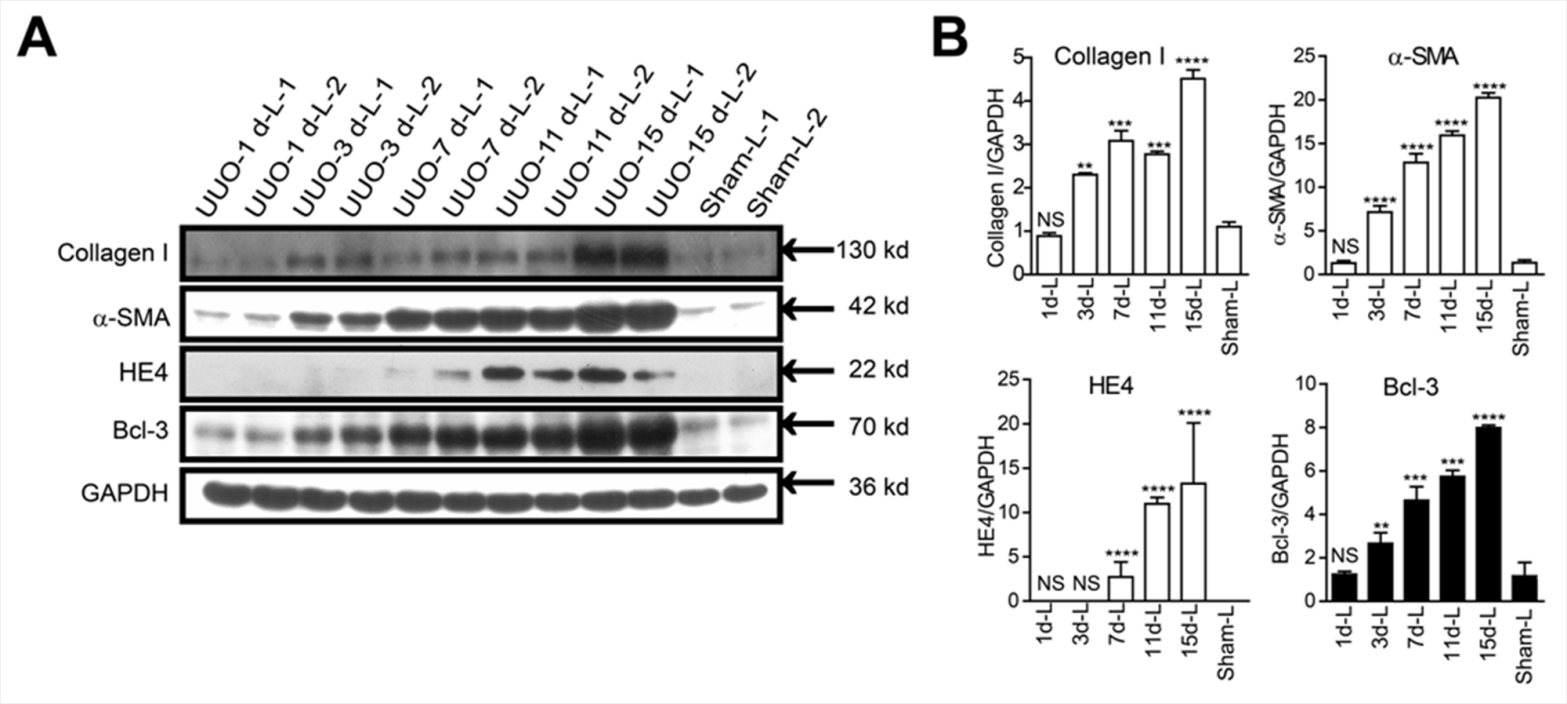
Bcl-3 protein level in fibrotic kidneys showed a significant increase from 3 days after UUO **(A)** Fibrotic markers (including type I collagen and α-SMA), HE4 and Bcl-3 expression levels were assessed using WB analysis in total kidney protein extracts. **(B)** Quantitative analysis of type I collagen, α-SMA, HE4 and Bcl-3 expression was obtained via densitometry analysis. All data are expressed as the means ± s.e.m. ^*^P < 0.05, ^**^P < 0.01, ^***^P < 0.001 and ^****^P < 0.0001 were determined using Student’s t-test. A total of 3 individuals were included in each sham group, and 5 individuals were included in each UUO group.

We ensured that all the surgeries were successfully conducted by assessing type I collagen and α-SMA protein levels. Similar to type I collagen and α-SMA protein, the amount of Bcl-3 protein significantly increased in the kidney lysates from mice ligated for 3 days and maintained at a high level until at 15 days after UUO surgery. In the meantime, we found the HE4 protein expression levels were up-regulated at 7 days after UUO surgery (Figure 4A and 4B).

### Bcl-3 expression was observed in the cytoplasm of renal tubular epithelial cells

**Figure 5:**
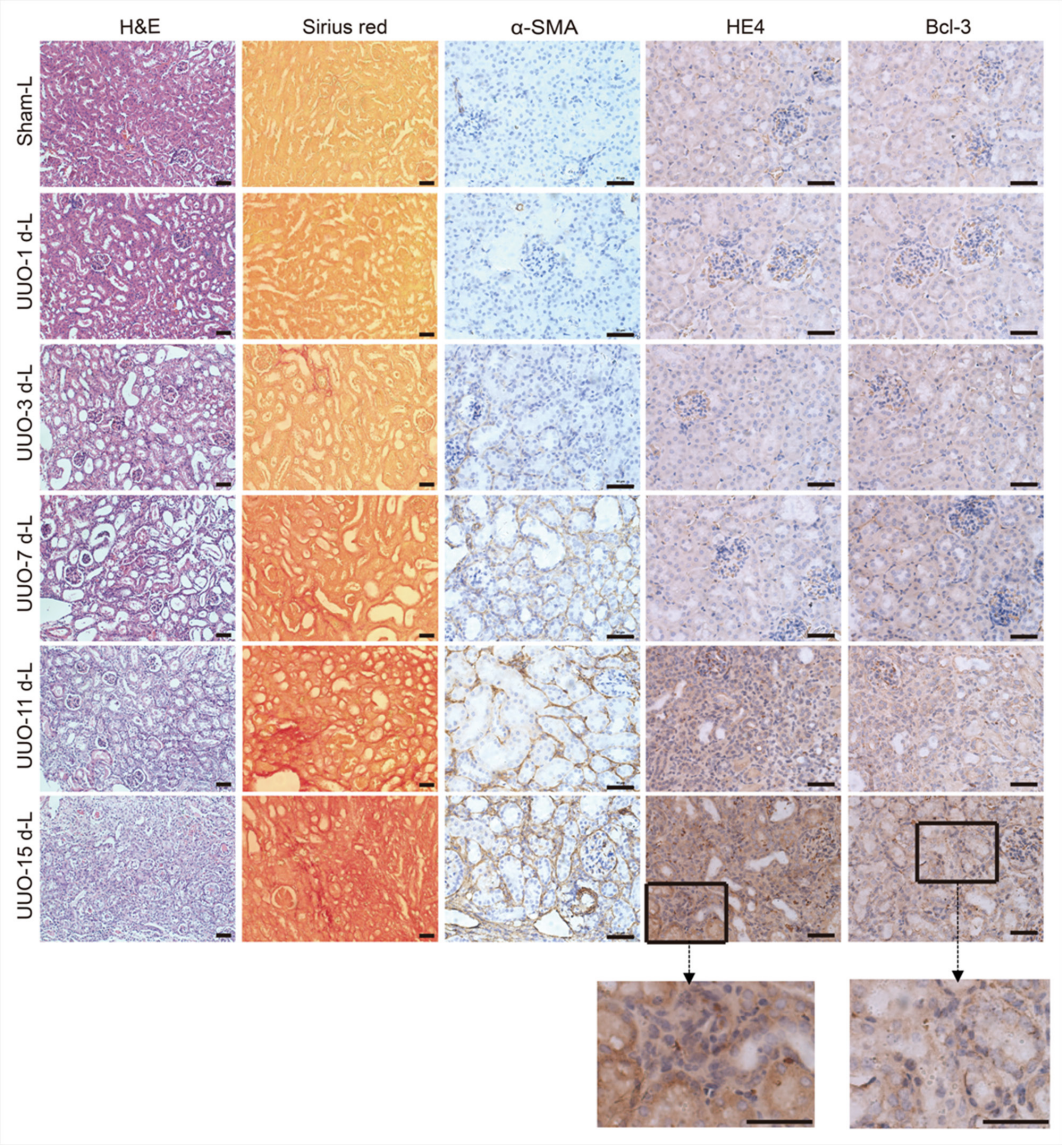
Bcl-3 expression was detected in the cytoplasm of renal tubular epithelial cells Representative H&E and Sirius red staining of control and fibrotic kidneys. Scale bars, 50 μm. Representative α-SMA images are presented, showing α-SMA protein expression levels. Scale bars, 50 μm. Representative HE4 images are presented, showing HE4 protein expression levels. Scale bars, 50 μm. Representative Bcl-3 images are presented, showing Bcl-3 protein expression levels. Scale bars, 50 μm.

In the UUO model, renal damage in the cortex of the obstructed kidney was shown by the H&E and type I collagen deposition was marked by Sirius red staining. We also observed more α-SMA-expressing cells. These data indicated that renal fibrosis was gradually induced. Both Sirius red staining and Immunohistochemistrical staining revealed that the accumulation of type I collagen, α-SMA and Bcl-3 protein was observed after only 3 days of obstruction, and large-scale deposition of these three proteins occurred on day 7 until day 15 after UUO surgery. In addition, significant HE4 protein deposition was not observed until day 7, and both HE4 and Bcl-3 expression was observed in the cytoplasm of renal tubular epithelial cells (Figure 5).

### Bcl-3 protein levels in serum were significant increased from 3 days after UUO

**Figure 6:**
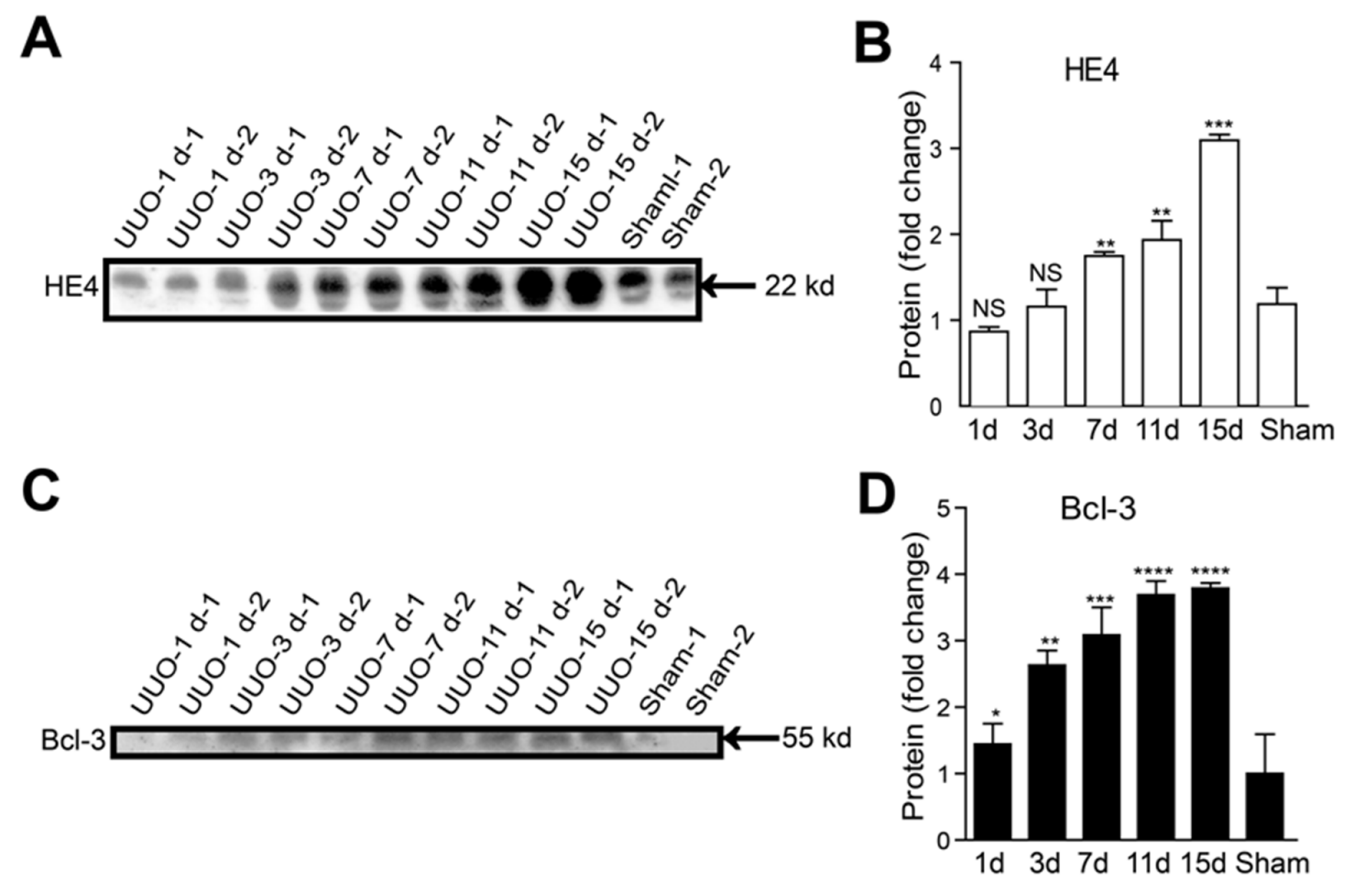
Serum Bcl-3 protein levels showed a significant increase from 3 days after UUO **(A, B)** The HE4 protein levels at different time points in UUO mice in 1 μL of serum per lane were detected using WB. Densitometry analysis of HE4 protein expression levels was conducted. **(C, D)** The Bcl-3 protein levels at different time points in UUO mice in 1 μL of serum per lane were detected using WB. Densitometry analysis of Bcl-3 protein expression levels was also performed. All data are expressed as the means ± s.e.m. ^*^P < 0.05, ^**^P < 0.01, ^***^P < 0.001 and ^****^P < 0.0001 were determined using Student’s t-test. A total of 3 individuals were included in each sham group, and 5 individuals were included in each UUO group.

HE4 has been demonstrated to be a valuable clinical biomarker of renal fibrosis in CKD [9, 10]. To explore whether Bcl-3 protein was released in the serum, we analyzed Bcl-3 serum levels in UUO mice (15 days after obstruction), Bcl-3 knockout mice and sham-operated mice using WB. Bcl-3 protein was detected in the sera of WT mice rather than those of Bcl-3 knockout mice. Serum Bcl-3 levels in the UUO model were higher than those in sham-operated mice (Supplementary Figure 1). To evaluate the value of Bcl-3 and HE4 served as biomarkers of renal fibrosis, we tested the levels of serum Bcl-3 and HE4 protein in the UUO model using western blotting due to a lack of reliable commercially available quantitative ELISA kits for mouse Bcl-3 and HE4. In the early stages of renal fibrosis, Bcl-3 protein levels were up-regulated prior to the up-regulation of HE4 protein levels (Figure 6A-6D), suggesting that although both Bcl-3 and HE4 protein levels in serum show strong positive correlations with renal fibrosis Bcl-3, as a potential biomarker, has a superior advantage and can serve as a biomarker for the diagnosis of renal fibrosis.

### Bcl-3 protein levels are significantly correlated with HE4 protein levels in the sera of healthy controls and CKD patients

**Figure 7:**
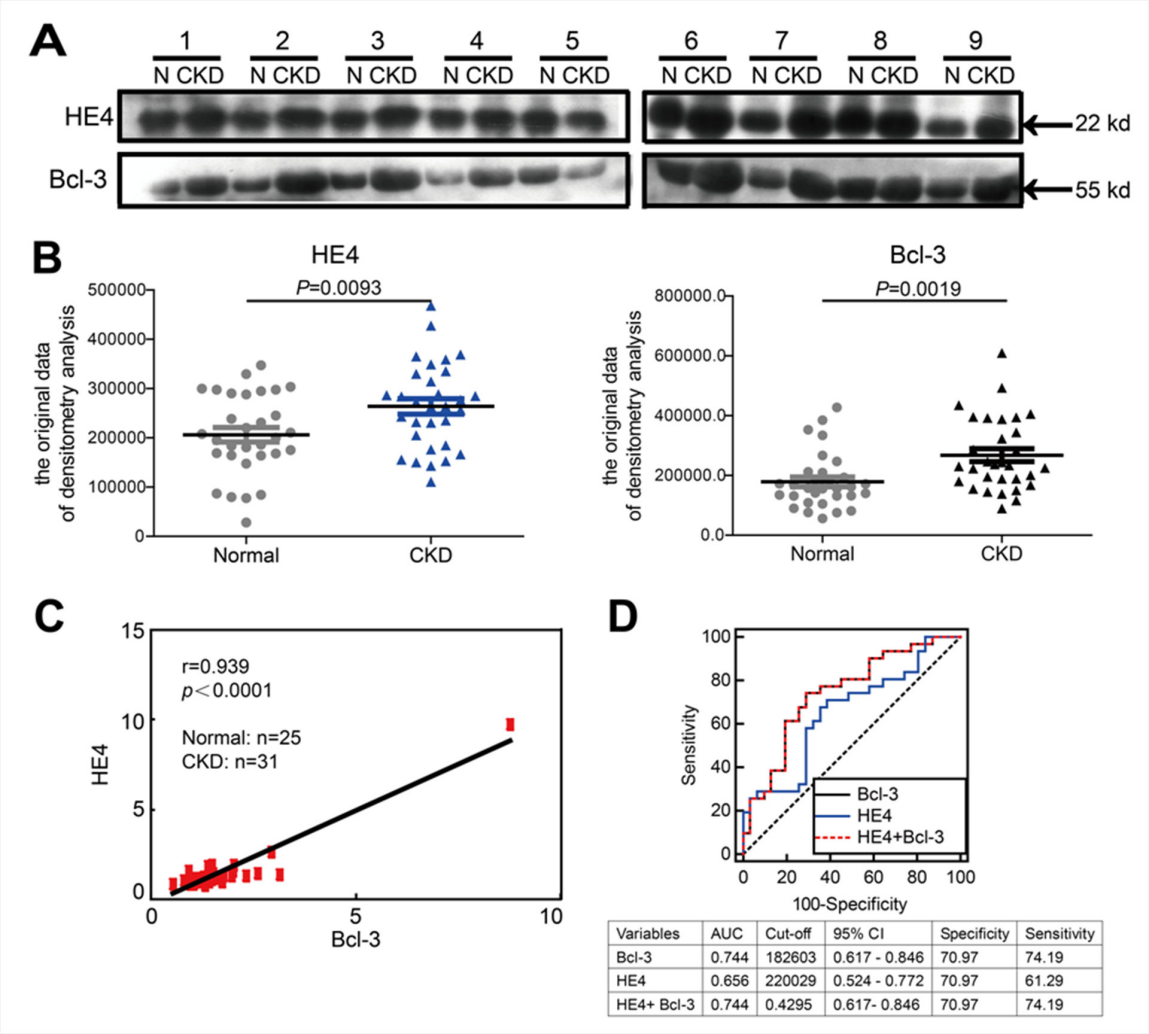
Bcl-3 protein levels are significantly correlated with HE4 protein levels in the sera of healthy controls and CKD patients **(A)** The serum protein levels of HE4 and Bcl-3 in 31 patients and normal controls were analyzed using WB, with 1 μl of serum per lane. Western blotting analysis of 9 healthy samples and 9 CKD patients is presented. **(B)** The blots were quantified using densitometry analysis. The serum protein levels of HE4 and Bcl-3 in 31 CKD patients were both higher than in the normal controls. **(C)** Pearson’s correlation coefficients analysis showed a strong positive correlation between HE4 and Bcl-3 (r = 0.939, *P* < 0.0001). **(D)** ROC analysis displayed the diagnostic power of serum HE4, Bcl-3 and combined markers in predicting renal fibrosis in CKD patients.

There were no obvious differences between patients and healthy subjects in terms of age or gender. The essential characteristics of the study participants are listed in Supplementary Table 1 and categorized according to renal function. Elevated serum concentrations of HE4 have recently been reported as a novel biomarker of disease severity and renal fibrosis in kidney disease. We examined the serum levels of HE4, creatinine, urea nitrogen, cystatin C and eGFR (glomerular filtration rate) was calculated. We found that consistent with the results previously reported [9], elevated serum HE4 was correlated with higher serum creatinine, urea nitrogen, serum cystatin C and lower eGFR. Pearson’s correlation coefficients between the two laboratory tests are listed in Supplementary Table 2. Due to a lack of reliable commercially available ELISA kits quantitating for human Bcl-3 protein, we analyzed Bcl-3 and HE4 protein levels in the sera of 31 CKD patients and 25 healthy controls using western blotting. Blots were further quantified by densitometry analysis. Consistent with the animal models, the protein levels of both Bcl-3 and HE4 in the sera of CKD patients were significantly higher than those in the sera of healthy controls (Figure 7A and 7B). Pearson’s correlation coefficients between HE4 and Bcl-3 showed a significant positive correlation between Bcl-3 and HE4 (r = 0.939, *P* < 0.0001) (Figure 7C). The predictive power of serum Bcl-3 and HE4 for detecting the severity of CKD patients was evaluated using receiver operating characteristics (ROC) analysis. The AUC-ROC (area under the ROC curve) of serum Bcl-3 was 0.7440 (*P*=0.00097) while the AUC-ROC of serum HE4 was 0.6556 (*P*=0.035). The ROC analysis of combined Bcl-3 and HE4 fully overlapped the Bcl-3 ROC analysis. ROC curve analysis indicated that Bcl-3 might be better than HE4 as a biomarker of renal fibrosis in CKD (Figure 7D). These results indicated that Bcl-3 could serve as a biomarker of renal fibrosis.

## DISCUSSION

Acute and chronic kidney injuries lead to of tubular epithelial cell death, associated with tubular atrophy and interstitial fibrosis. In the progression of renal fibrosis, the unconstrained TGFβ/Smad signaling is considered to be the most important pathway. In addition, the inflammatory factors released through cell death may induce the production of Bcl-3, which further regulates the TGFβ signaling pathway. In the present study, Bcl-3 was markedly up-regulated in the early stages of renal fibrosis in the UUO mouse model. In the patients with CKD the serum Bcl-3 level was significantly higher than in healthy controls and was highly correlated with the serum level of HE4, a recognized biomarker for renal fibrosis and renal dysfunction. These data reveal that Bcl-3 might be a novel biomarker of renal fibrosis in CKD.

Bcl-3 induction in the renal tissues of the UUO mice appeared in the early stage of renal fibrosis, similar to other fibrosis biomarkers, including *Wfdc2, Snai1, Acta2, Col1α1, Tgfb1, P21* and *Twist1*. We observed that Bcl-3 and HE4 were highly expressed in the cytoplasm of liberal epithelial cells in the renal tissue of UUO mice. Thus far, the stimuli inducing Bcl-3 expression in renal tubular epithelial cells remained unclear. Bcl-3 is typically located in the cytoplasm and nucleus of most cells. We used western blotting to detect the levels of Bcl-3 protein in the sera of UUO mice. Bcl-3 protein was detectable in the sera of Bcl-3 WT mice, but not in the sera of Bcl-3 KO mice (Supplementary Figure 1), and serum Bcl-3 protein levels gradually increased with the progression of renal fibrosis. These results confirmed that Bcl-3 protein was really released into and existed in the sera of UUO mice. The increased Bcl-3 protein in the sera may reflect its release to sera from apoptotic cells after injury. Indeed, Bcl-3 protein was also detected in the supernatant of tumor cells expressing high levels of Bcl-3 (data not shown), suggesting that Bcl-3 may also be induced during renal fibrosis and autonomously released into the serum in the exosome pellet.

Bcl-3 mRNA and protein levels were increased prior to HE4 mRNA and protein, in the renal tissues of UUO mice. Bcl-3 protein was detectable and increased in patients with CKD and correlated with serum HE4 protein. These data showed that in addition to HE4, Bcl-3 might be a novel biomarker of renal fibrosis in patients with CKD. The ROC analysis suggested that Bcl-3 was better than HE4 as a biomarker of renal fibrosis. Serum levels of Bcl-3 protein increased earlier than serum HE4 in the UUO mouse model, suggesting that Bcl-3 was more sensitive than HE4 as a biomarker of renal fibrosis. Although the combination of serum Bcl-3 and HE4 was not better than Bcl-3 alone using western blotting in CKD patients, a quantitative measure of serum Bcl-3 and HE4 is needed to re-evaluate the value of the combination of Bcl-3 and HE4 in the detection of renal fibrosis in CKD patients.

We and others have reported that Bcl-3 mRNA and protein levels are increased in the tumor tissues compared with normal tissues in various types of cancer including colorectal cancers, breast cancers [5, 16, 23]. Whether serum Bcl-3 protein levels in the patients with cancers are increased needs to be further investigated. Nevertheless, we provided the evidence that Bcl-3 could serve as a novel biomarker of renal fibrosis to monitor the disease progression in CKD, particularly in combination with other markers such as HE4 in the non-cancerous patients.

Due to a lack of reliable commercially available quantitative ELISA kits, Bcl-3 protein in the sera was semi-quantitatively detected using western blotting which usually reflects Bcl-3 protein levels more specifically. Thus, the quantitative examination of serum Bcl-3 protein in additional CKD patients is needed to determine the diagnostic significance of Bcl-3 in renal fibrosis. Although the data in the present study showed a significant correlation between serum Bcl-3 and serum HE4 protein levels, the evaluation of renal fibrosis in renal biopsies and the quantitative examination of serum Bcl-3 protein and HE4 protein in patients with CKD are required to confirm the value of Bcl-3 and its combination with HE4 in the detection of renal fibrosis.

In conclusion, we demonstrated that elevated levels of serum Bcl-3 were associated with decreased kidney function. The results of this study suggest that Bcl-3 can be a valuable clinical biomarker of renal fibrosis in CKD.

## MATERIALS AND METHODS

### Mouse model of UUO

All animals were housed and maintained under specific pathogen-free conditions. All animal experiments were performed in compliance with the guidelines for the care and use of laboratory animals and were approved by the Institutional Biomedical Research Ethics Committee of the Shanghai Institutes for Biological Sciences at the Chinese Academy of Sciences. Six- to 8-week-old male C57BL/6 mice were supplied by Shanghai Laboratory Animal Center, Chinese Academy of Sciences, Shanghai. The mice underwent ligation of the left ureter. Prior to surgery the mice were anesthetized with 5% sodium pentobarbital (30 mg/kg). The left ureter was either completely obstructed at 1 cm below the renal pelvis using 4.0 silk ligature (ligated animals) or similarly manipulated but not ligated (sham-operated animals). Subsequently, the kidneys were collected, rinsed with PBS, dissected and stored in liquid nitrogen until further analysis.

### Real-time PCR analysis

RNA was isolated from kidneys tissue using TRIzol Reagent (Invitrogen, Cat. 15596-018), reverse transcription was performed using Transcript First Strand Synthesis Supermix (TransGen Biotech, Cat. AT301), according to the manufacturer’s instructions. All qRT-PCRs were performed using a 7900 Fast Real-Time PCR System (Applied Biosystems), and SYBR Green PCR Master mix was purchased from Applied Biosystems (Applied Biosystems, Cat. 43-676-59). Each sample was analyzed in triplicate or greater replicates. Relative quantification was derived from the difference in the cycle threshold (Ct) between the target gene and GAPDH (ΔCt) and compared with control cell lines using the equation RQ=2-ΔΔCt. Error bars represented the standard deviation (SD), and the significance of any differences was calculated using a two-tailed, unpaired t-test.

### Western blotting analysis

Western blotting was carried out using standard procedures with the following primary antibodies (suppliers): anti-type I collagen (1:1000 dilution, ProteinTech Group, Cat. 14695-1-AP), anti-α-SMA (1:1000 dilution, Abcam, Cat. ab5694), anti-Bcl-3 (1:200 dilution, Santa Cruz Biotechnology, Cat. sc-185), anti-HE4 (1:1000 dilution, ProteinTech Group, Cat. 14406-1-AP), anti-GAPDH (1:3000 dilution, ProteinTech Group, Cat. HRP-60004).

### Histology and immunohistochemistry

Kidney tissues were fixed with 4% PFA in PBS for 24 h and embedded in paraffin. 5 μm sections were cut using a paraffin microtome with stainless steel knives. The sections were mounted on glass slides, deparaffinized with xylene, dehydrated through a graded series of ethanol and stained with hematoxylin-eosin. To evaluate collagen deposition, sections were stained with Sirius red (saturated aqueous solution of picric acid containing 0.1% Direct Red80) (Sigma-Aldrich, Cat. 365548). IHC analyses of diaminobenzidine staining were performed using an HRP kit (ScyTek, Cat. Scytek-UHP500). The specimens were visualized using a general optical microscope with a camera (Carl Zeiss). Images were processed with equivalent parameters using ZEN Light Edition software (Carl Zeiss).

### Clinical characteristics

The study participants were recruited in the Chinese People’s Liberation Army 105th Hospital from June 2014 to July 2015. The 31 CKD patients were inpatients at the Department of Clinical Laboratory of the Chinese People’s Liberation Army 105th Hospital because of continued proteinuria, on-going decline in eGFR levels, or dialysis. The normal subjects group included 25 age-matched healthy controls with normal renal function (eGFR > 90 mL/min/1.73 m2). The ethics committee of the Chinese People’s Liberation Army 105th Hospital has approved the study according to Declaration of Helsinki guidelines. All participants enrolled in this study gave their written informed consent. HE4 was tested by ARCHITECT i2000 HE4 assay (Abbott Diagnostics) according to the manufacturer’s instructions. Serum cystatin C, creatinine and urea nitrogen were all measured by ADVIA2400 (Siemens) according to the manufacturer’s instructions. Glomerular filtration rates (eGFR) were calculated based on the age, gender and serum creatinine concentrations of individuals.

## SUPPLEMENTARY MATERIALS FIGURE AND TABLES



## Abbreviations

CKD: chronic kidney disease
UUO: unilateral ureteral obstruction
Bcl-3: B cell lymphoma 3
HE4: human epididymis protein 4
TGFβ: transforming growth factor β
NFκB: nuclear factor-κB
ECM: extra cellular matrix
Col1a1: type I collagen
IκB: NFκB inhibitor
WT: wide-type
α-SMA: α-smooth muscle actin
EMT: epithelial-to-mesenchymal transition
GFR: glomerular filtration rate
ROC: receiver operating characteristic
