# Supplementary material for: Bcl-3 is a novel biomarker of renal fibrosis in chronic kidney disease
Source: Oncotarget. 2017 Oct 9;8(57):97206–16. doi: 10.18632/oncotarget.21692 (PMC5722556; doi:10.18632/oncotarget.21692)
Supplement: Supplementary file 1 [file oncotarget-08-97206-s001.pdf]

Bcl-3 is a novel biomarker of renal fibrosis in chronic kidney disease

SUPPLEMENTARY MATERIALS

Supplementary Table 1: Clinical characteristics and laboratory tests for all 56 individuals

Variables	Controls (n=25)	CKD2 (n=9)	CKD3 (n=12)	CKD4 (n=6)	CKD5 (n=4)
Age (year)	52.4±20.4	58.0±32.0			
	P=0.067				
Gender (M/F)	25 (14/11)	31(18/13)			
eGFR (mL/min/1.73 m^2)	-	49.8 (2.3-84.1)			
cystatin C (mg/L)	-	2.5 (0.6-9.0)			
creatinine (μmol/L)	-	203.4 (39.0-1372.0)			
Urea nitrogen (mmol/L)	-	10.7 (2.1-35.5)			
HE4 (pmol/L)	49.2(32.2-93.4)	372.0 (39.1-1500)			

The essential characteristics of the 56 study participants are shown here, describing renal function, including eGFR, cystatin C, creatinine, urea nitrogen and HE4.

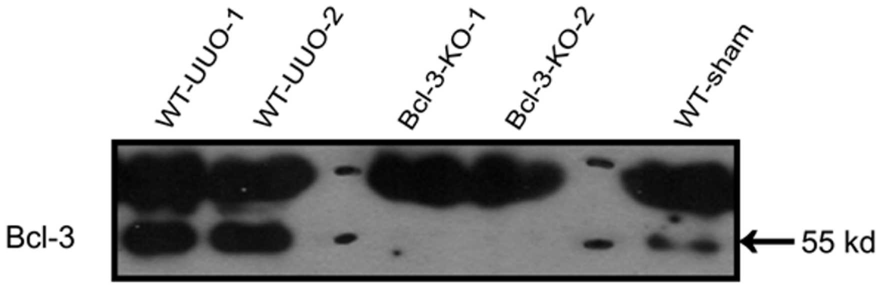

Supplementary Figure 1: Bcl-3 protein was undetectable in the sera of Bcl-3 KO mice. The Bcl-3 protein levels in the sera of UUO mice (15 days after obstruction), Bcl-3 KO mice and sham-operated mice were examined using western blotting.

Supplementary Table 2: Clinical characteristics and laboratory tests for all 56 individuals

	Age	creatinine	eGFR	Urea nitrogen	cystatin C
HE4					
r	0.1187	0.7287	-0.6138	0.6351	0.8927
p	0.5249	<0.0001	0.0002	0.0001	<0.0001
Age					
r	1	0.0809	-0.3310	0.0865	0.2465
p	-	0.6654	0.0689	0.6435	0.2249
creatinine					
r		1	-0.5584	0.7442	0.6700
p		-	0.0011	<0.0001	0.0002
eGFR					
r			1	-0.5600	-0.6570
p			-	0.0011	0.0003
Urea nitrogen					
r				1	0.5371
p				-	0.0047
cystatin C					
r					1
p					-

The correlation and *P*-value between the two tests were analyzed by Pearson's correlation coefficients.
